# A Four-Stepwise Electrocardiographic Algorithm for Differentiation of Ventricular Arrhythmias Originated from Left Ventricular Outflow Tract

**DOI:** 10.3390/jcm11216398

**Published:** 2022-10-28

**Authors:** Hong-Wei Tan, Wei-Dong Gao, Xin-Hua Wang, Zhi-Song Chen, Xue-Bo Liu

**Affiliations:** 1 Department of Cardiology, Tongji Hospital, Tongji University School of Medicine, No. 389 Xincun Road, Shanghai 200065, China; 2 Department of Cardiology, Jiangmen Central Hospital, Jiangmen 529030, China; 3 Department of Cardiology, Ren Ji Hospital, School of Medicine, Shanghai Jiao Tong University, 1630 Dongfang Rd., Shanghai 200127, China

**Keywords:** ventricular arrhythmias, catheter ablation, electrocardiogram, algorithm, left ventricular outflow tract

## Abstract

Several electrocardiographic algorithms have been proposed to identify the site of origin for the ventricular arrhythmias (VAs) from the left ventricular outflow tract (LVOT) versus right ventricular outflow tract. However, the electrocardiographic criteria for distinguishing VAs originated from the different sites of LVOT is lacking. We aimed to develop a simple and efficient ECG algorithm to differentiate LVOT VAs originated from the aortic root, AMC and LV summit. We analyzed 12-lead ECG characteristics of 68 consecutive patients who underwent successful radiofrequency catheter ablation of symptomatic VAs from LVOT. Patients were divided into RCC (right coronary cusp) group (*n* = 8), the L-RCC (the junction between the LCC and RCC) group (*n* = 21), the LCC (left coronary cusp) group (*n* = 24), the aortomitral continuity (AMC) group (*n* = 9) and the LV summit group (*n* = 6) according to the final ablation sites. Measurements with the highest diagnostic performance were modeled into a 4-stepwise algorithm to discriminate LVOT VAs. The performance of this novel algorithm was prospectively tested in a validation cohort of 43 consecutive patients undergoing LVOT VAs ablation. Based on the accuracy of AUC, a 4-stepwise ECG algorithm was developed. First, the QS duration in aVL > 134 ms was used to distinguish VAs from AMC, LV summit and VAs from aortic root (80% sensitivity and 76% specificity). Second, the R duration in II > 155 ms was used to differentiate VAs from LV summit and VAs from AMC (67% sensitivity and 56% specificity). Third, the ratio of III/II < 0.9 was used to discriminate VAs from RCC and VAs from LCC, L-RCC (82% sensitivity and 63% specificity). Fourth, the QS duration of aVR > 130 ms was used to discern VAs from LCC and VAs from L-RCC (75% sensitivity and 62% specificity). In the prospective evaluation, our 4-stepwise ECG algorithm exhibited a good predictive value. We have developed a novel and simple 4-stepwise ECG algorithm with good predictive value to discriminate the AVs from different sites of LVOT.

## 1. Introduction

Idiopathic ventricular arrhythmias (VAs) often originate from the left ventricular outflow tract (LVOT) [1,2,3,4], and up to one third of all VAs are thought to arise from the LVOT region [5]. Anatomically, LVOT lies in the center of the heart and includes the aortic root, aortomitral continuity (AMC), the superior basal septum and LV summit [3]. LVOT VAs can be successfully treated by endocardial radiofrequency catheter ablation (RFCA) from the aortic root, AMC or from the LV summit via cardiac venous system [3,4]. The surface electrocardiogram (ECG) is a simple and relatively accurate evaluation tool for localization of VAs before ablation. The electrocardiographic characteristics of VAs originating from the aortic root, AMC and LV summit have been studied [3,4,5], and several ECG criteria have been proposed to identify the site of origin for the VAs from the LVOT versus right ventricular outflow tract [6,7,8,9,10]. However, the electrocardiographic criterion for distinguishing VAs originating from the different sites of LVOT is still lacking. The aim of our study was to develop a simple and efficient ECG algorithm to differentiate LVOT VAs that originated from the aortic root, AMC and LV summit.

## 2. Methods

### 2.1. Study Design 

This study was designed in 2 phases: (1) a retrospective analysis of successful LVOT VAs ablation cases in order to develop an ECG algorithm; and (2) a prospective cohort study to verify the accuracy and effectiveness of our ECG algorithm.

### 2.2. Study Populations

Consecutive patients with symptomatic LVOT VAs who were successfully ablated from January 2019 to December 2020 were included in this study. LVOT VAs were defined as VAs with inferior axis, a R/S transition in lead V3 or earlier and a site of the earliest ventricular activation in the aortic root, AMC and LV summit [11]. Physical examination, 12-lead ECG, 24-h Holter monitor, and echocardiography demonstrated no evidence of structural heart disease in any patients. Baseline characteristics, including age, gender, nature of the clinical arrhythmia, and 12-lead ECG during the VAs, were recorded. All antiarrhythmic drugs were discontinued for at least five-lives before the study. The Institutional Review Board approved the study protocol, and informed consent for the procedure was obtained from all the patients.

### 2.3. Electrophysiological Study

All patients underwent electrophysiological examination in the postabsorptive, nonsedated state. A catheter was placed in the right ventricle, or within the distal coronary sinus, via femoral veins if necessary. Surface ECG leads were applied in the standard manner. ECG and intracardiac electrograms were recorded simultaneously by digital multichannel system (EPMed, St Jude Medical or Pruca Cardiolab, GE Healthcare), filtered at 30 to 500 Hz for bipolar electrograms and at 0.05 to 400 Hz for unipolar electrograms. If VAs were not inducible at baseline, intravenous isoproterenol infusion (2–5 ug/min) was administrated to induce the clinical VAs. If clinical arrhythmia failed to occur spontaneously, programmed stimulation was performed. The standard protocol consisted of ventricular stimulation at 2 basic drive cycle length with ≤2 extrastimuli to a minimum coupling interval of 230 ms as described previously [12]. Mapping and pacing were performed using a 7.5Fr, 3.5-mm-tip irrigated ablation catheter (Navistar ThermoCool; Biosense Webster, Diamond Bar, CA, USA) introduced from the right femoral vein for sites in the right ventricular outflow tract and LV summit or right femoral artery for the endocardial LVOT and AMC. Intravenous heparin was administered to maintain an activated clot time > 250 s when mapping at the aortic root and left ventricle. 

### 2.4. Mapping and Radiofrequency Catheter Ablation

The procedure was performed under the guidance of fluoroscopy and the 3-dimensional electroanatomic mapping system (CARTO 3, Biosense Webster, Diamond Bar, CA). In patients with frequent premature ventricular contractions (PVC), activation mapping was performed in the right ventricular outflow tract, the aortic root, AMC and great cardiac veins. In some cases, the area underneath aortic sinus cusps was reached by an antegrade trans-septal approach as described previously [12]. When PVC was infrequent, pace mapping was performed during sinus rhythm at a pacing cycle length of 500 ms at an output just greater than a diastolic threshold, as previously described [8,13]. The ablation target was defined as the earliest bipolar electrogram preceding the QRS onset during the PVC and/or an excellent pace map (>11/12 leads) [8]. When the earliest ventricular activation at the intended local site preceded QRS onset by >20 ms [14] and/or excellent pace mapping was achieved, RFCA was performed in temperature-controlled mode with a maximum power of 35 W, temperature limit of 43 °C, and flush rate of 20 mL/min in aortic root, AMC and with a maximum power of 30 W, temperature limit of 43 °C, and flush rate of 30 mL/min in great cardiac veins. If the impedance was too high (more than 200 Ω, even more), we usually flush the catheter first and wait for the impedance to decrease to less than 180 Ω, then we begin ablation. If the earliest ventricular activation site was identified in RVOT or ablation was performed in RVOT, the patients were excluded from the study. If the VAs require multiple sites (≥2) ablation, the cases were also excluded.

Coronary angiography was performed to assess the course of left and right coronary artery and catheter ablation from the aortic root/coronary cusp; this was only performed if the distance from the electrode tip to the ostium of each left and right coronary ostium was >8 mm as detected by simultaneous coronary angiography [4]. Radiofrequency energy application from the great cardiac veins was performed at sites where the distance to the adjacent coronary artery was >5 mm, as determined by simultaneous coronary angiography [4]. During radiofrequency energy delivery, if a decreased frequency/ elimination of VAs occurred within the initial 15 s, the radiofrequency delivery was continued for 30 to 60 s. Otherwise, the energy delivery was terminated, and the ventricular arrhythmias were re-mapped. Successful ablation was defined as the absence of spontaneous or inducible VAs with isoproterenol infusion (2–5 ug/min) and burst pacing from the RV or LV 30 min after the final radiofrequency application. 

### 2.5. ECG Analysis 

The surface 12-lead ECG were recorded during sinus rhythm and during the VAs at a sweep speed of 100 mm/s for all patients. The electrodes of leads V1 and V2 were placed at the fourth intercostal space with careful attention to minimize the effect of incorrect electrode placement on the QRS morphology [15].The following parameters were measured during clinical VAs: (1) total QRS duration; (2) R wave duration and amplitude of inferior leads (II, III, aVF) and the R wave amplitude ratio of lead III/II; (3) QS wave duration and amplitude in leads aVL and aVR and the QS wave amplitude ratio of aVL/aVR; (4) R and S wave amplitude in leads V1-V6; and (5) the site of R wave transition in the precordial leads. The T-P segment was considered the isoelectric baseline for measurement of R wave and S wave amplitudes. The total QRS duration was measured from the site of earliest initial deflection from the isoelectric line in any lead to the time of latest activation in any lead. The R-wave duration was measured from the site of the earliest initial deflection from the isoelectric line to the time at which the R-wave intersected the isoelectric line [16]. For all cases, QRS measurements were performed before mapping and ablation. The ECG analysis was performed by two experienced investigators blinded to the site of the origin with electronic calipers. 

### 2.6. Procedure Success and Follow-Up 

Acute success was defined as absence of spontaneous or provoked clinical VAs at 30 min after the last radiofrequency energy application. Follow-up was performed by referring physicians or outpatient clinics.

### 2.7. Statistical Analysis

Continuous variables are expressed as mean ± SD. Analysis of variance (ANOVA) followed by the Tukey test was used to compare the difference among groups. Categorical variables were compared by a chi-squared test analysis or Fisher’s exact test. For those ECG measurements that were significantly different between the anatomical sites, a receiver operating characteristic (ROC) curve was performed to determine cut-offs with optimal performance. These were then subjected to stepwise mechanistic analysis in varying order and combination to derive an algorithm with optimal statistical accuracy and the sensitivity, specificity, positive predictive value (PPV), and negative predictive value (NPV) for predicting the origins of VAs were assessed using the standard formula. All statistical analyses were performed using SPSS 15.0 software (SPSS Inc., Chicago, IL, USA), and the differences were considered significant at *p* < 0.05.

## 3. Results

### 3.1. Patient Characteristics

The study population consisted of 68 patients (28 men, mean age 54 ± 17 years [range, 17–85]) with symptomatic idiopathic sustained ventricular tachycardia (VT; *n* = 2, non-sustained VT (*n* = 5), or premature ventricular contractions (PVCs; *n* = 61). The VAs origin was identified in the LVOT by 3-dimensional mapping and a successful catheter ablation. The patients were further divided into five groups according to the final ablation site which includes the RCC (right coronary cusp) group (*n*= 8), the L-RCC (the junction between the LCC and RCC) group (*n* = 21), the LCC (left coronary cusp) group (*n* = 24), the AMC group (*n* = 9) and the LV summit group (*n* = 6). No structural heart disease was found on physical examination, 12-lead ECG or echocardiography. All patients were refractory to ≥1 antiarrhythmic drug before ablation. The demographic and clinical data are shown in Table 1. There was no significant difference in terms of age, gender, diameter of LV, burdens of VAs and comorbidity. Patients in LV summit groups showed significant longer VAs history than patients in RCC and L-RCC groups.

### 3.2. Mapping and Ablation 

All patients underwent electrophysiological study and 3-dimensional mapping. The successful ablation site was located in the RCC in 8 patients, L-RCC in 21, LCC in 24, AMC in 9 and LV summit in 6 patients. There were no significant differences in local ventricular activation time relative to QRS onset at the successful ablation sites, or duration of radiofrequency application among groups. Compared to other groups, all patients in the LV summit group demonstrated larger A wave at the ablation site.

### 3.3. ECG Analysis

All patients showed R morphology in inferior leads and QS morphology in aVR and aVL. As for the precordial leads, all the patients demonstrated a R/S transition in lead V3 or earlier. Patients in AMC and LV summit groups demonstrated Rs morphology in lead V1 while patients with VAs from aortic root showed rs or rS morphology in lead V1.

**Figure 1:**
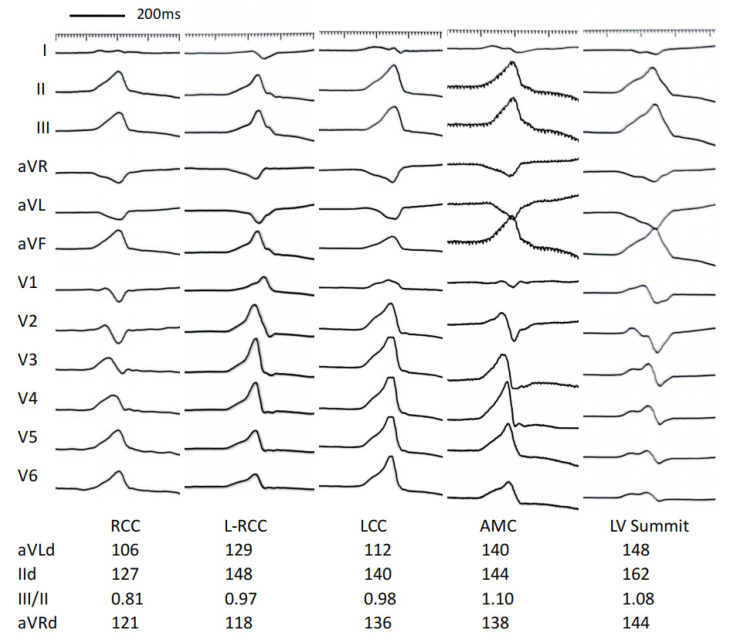
Examples of the electrocardiographic morphology of ventricular arrhythmias originating from different sites of LVOT. aVLd, The QS duration in aVL; IId, The R duration in II; III/II, The ratio of III/II; aVRd, The QS duration in aVR.

The results of the electrocardiographic parameters of the VAs are showed in Table 2 and Table 3 and Figure 1. The total QRS duration was significantly increased in the LV summit group than in other groups (*p* < 0.05 or *p* < 0.01) and was significantly increased in the LV summit group than in the AMC group (*p* < 0.05). The R wave duration in lead II, III and aVF was significantly increased in the LV summit group than in RCC, L-RCC, and LCC groups (all *p* < 0.01), and was significantly increased in the AMC group than in the RCC and L-RCC groups (all *p* < 0.01). The R wave duration in lead II showed significant difference between LV summit group and AMC group (*p* < 0.05). The QS duration in lead aVL and aVR was significantly increased in the LV summit group than in VAs from aortic root (all *p* < 0.01), and was significantly increased in the AMC group than in VAs from RCC and L-RCC groups (all *p* < 0.01). The QS duration in lead aVL was significantly increased in the AMC group compared to VAs from LCC (*p* < 0.01). 

The R wave amplitude in Lead III and aVF was significantly higher in VAs localized in LCC compared to VAs originated from RCC (*p* < 0.01). Compared to other groups, the QS wave amplitude in lead aVL was significantly decreased in the RCC group (all *p* < 0.01). The QS wave amplitude in lead aVR was significantly decreased in the AMC group than in the L-RCC and LCC groups (all *p* < 0.01).

The R wave amplitude ratio of lead III/II was significantly decreased in the RCC group compared to VAs from other groups (all *p* < 0.01). The R wave amplitude ratio of lead III/II was significantly decreased in the L-RCC group than in the AMC group (*p* < 0.01). The QS wave amplitude ratio of aVL/aVR was significantly decreased in the RCC group compared to VAs from other groups (all *p* < 0.01). The QS wave amplitude ratio of aVL/aVR was significantly decreased in the L-RCC group than in the AMC group (*p* < 0.01).

In the precordial leads, the R wave duration and amplitude in Lead V1 and V2 demonstrated a progressive increment with significant difference between VAs from AMC, LV summit and VAs from aortic root, while the S wave duration and amplitude showed a tendency to decrease from aortic root to AMC and LV summit groups.

### 3.4. Develop a Stepwise ECG Algorithm to Differentiate LVOT VAs 

**Figure 2:**
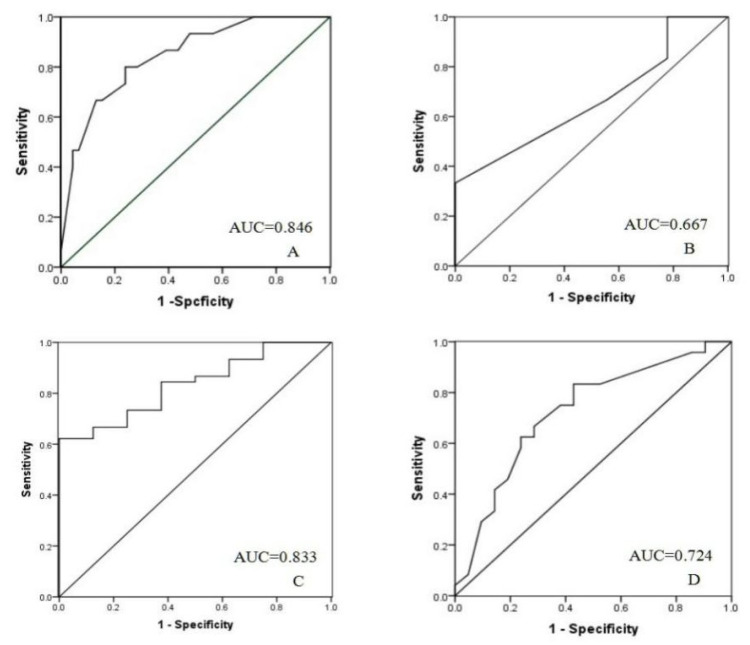
Receiver operating characteristic (ROC) curve of the QS duration in aVL (**A**), the R duration in II (**B**), the ratio of III/II (**C**) and the QS duration in aVR (**D**) to distinguish LVOT VAs.

The ability of ECG parameters to distinguish LVOT VAs was assessed by using an ROC curve. The area under the curve for the ROC curve of the QS duration in aVL was 0.846 [95% confidential interval 0.735–0.956]. The QS duration in aVL >134 ms had 80% sensitivity and 76% specificity for differentiating VAs from AMC, LV summit and VAs from aortic root. The area under the curve for the ROC curve of the R duration in II was 0.667 [95% confidential interval 0.369–0.965]. The R duration in II > 155 ms had 67% sensitivity and 56% specificity for differentiating VAs from AMC and VAs from LV summit. The area under the curve for the ROC curve of the ratio of III/II was 0.833 [95% confidential interval 0.711–0.955]. The ratio of III/II < 0.9 had 82% sensitivity and 63% specificity for differentiating VAs from RCC and VAs from L-RCC, LCC. The area under the curve for the ROC curve of the QS duration in aVR was 0.724 [95% confidential interval 0.573–0.876]. The QS duration in aVR > 130 ms had 75% sensitivity and 62% specificity for differentiating VAs from LCC and VAs from L-RCC (Figure 2). 

**Figure 3:**
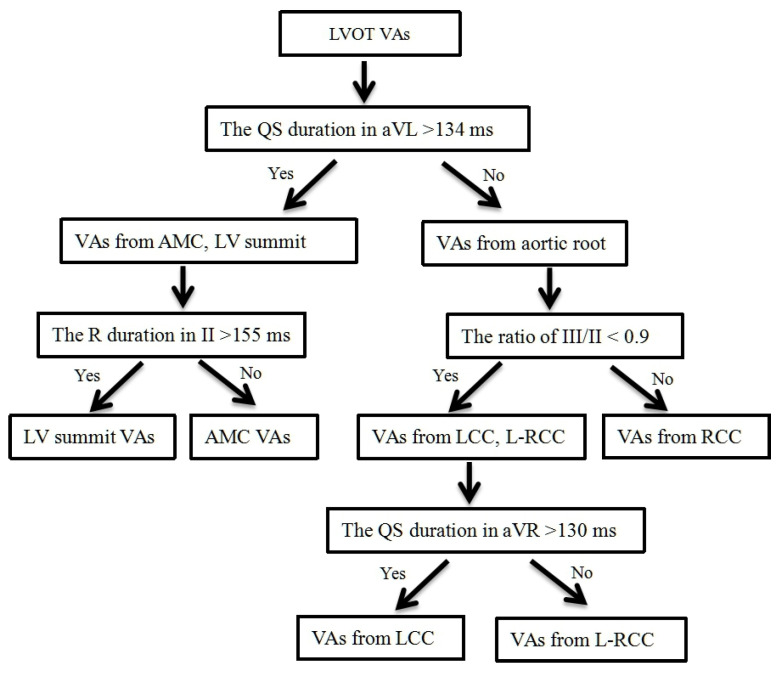
Flowchart of 4-stepwise electrocardiographic algorithm.

According to the accuracy of AUC, a stepwise ECG algorithm was developed (Figure 3). First, QS duration in aVL > 134 ms was used to distinguish VAs from AMC, LV summit and VAs from aortic root. Second, R duration in II > 155 ms was used to differentiate VAs from LV summit and VAs from AMC. Third, the ratio of III/II < 0.9 was used to discriminate VAs from RCC and VAs from LCC, L-RCC. Fourth, the QS duration of aVR > 130 ms was used to discern VAs from LCC and VAs from L-RCC.

### 3.5. Validation of Four-Stepwise ECG Algorithm in the Prospective Study

**Figure 4:**
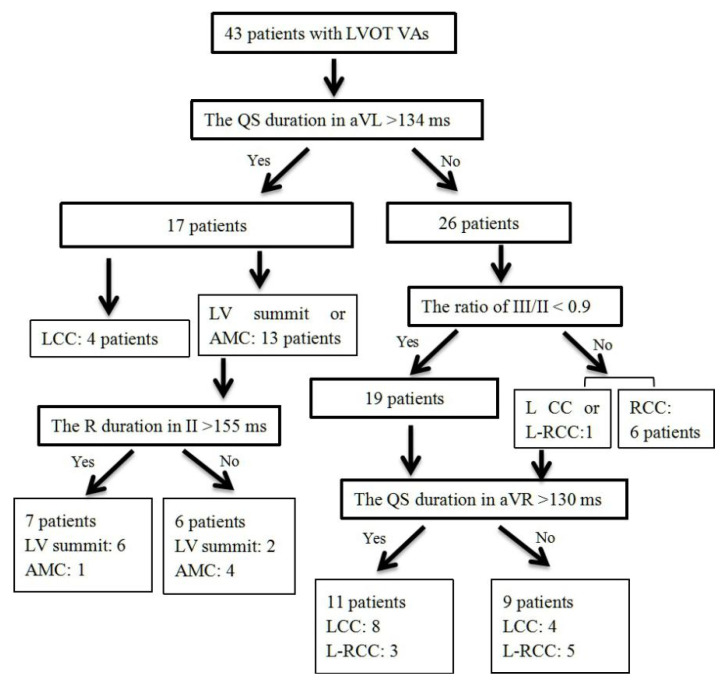
Process of validation in the prospective cohort patients.

A total of 43 consecutive patients (22 males and 21 females) with a left branch block pattern and inferior axis QRS morphology, and with precordial transition before lead V3 that indicate VAs from LVOT, were selected to verify the effectiveness of our four-stepwise algorithms. All these patients underwent successful RFCA for VAs between January 2021 and December 2021 at the sites of LVOT. The successful ablation site was located in the RCC in 6 patients, L-RCC in 8, LCC in 16, AMC in 5 and LV summit in 8 patients. The QS duration in aVL > 134 ms exhibited 100% sensitivity, 86.7% specificity, 76.5% positive predictive value and 100% negative predictive value for differentiating VAs from AMC, LV summit and VAs from aortic root. The R duration in II > 155 ms had 75.0% sensitivity, 80.0% specificity, 85.7% positive predictive value and 66.7% negative predictive value for differentiating VAs from AMC and VAs from LV summit. The ratio of III/II < 0.9 showed 100% sensitivity, 95.0% specificity, 85.7% positive predictive value and 100% negative predictive value for distinguishing VAs from RCC and VAs from LCC, L-RCC. The QS duration in aVR > 130 ms showed 66.7% sensitivity, 62.5% specificity, 72.7% positive predictive value and 55.6% negative predictive value for differentiating VAs from LCC and VAs from L-RCC (Table 4, Figure 4).

## 4. Discussion

This study explored the surface ECG characteristics of VAs originating from LVOT and we present a simple four-stepwise ECG algorithm to differentiate VAs from different sites of LVOT. We have demonstrated that in patients with VAs when ECG strongly favors an LVOT origin, our four-stepwise ECG algorithm can help localize the likely sites of origin during planning for VAs ablation. First, AMC and LV summit VAs can be differentiated from aortic root VAs by measuring the QS duration in aVL. Then, differentiation between AMC and LV summit VAs can be made by the R duration in II. Third, the ratio of III/II was used to distinguish VAs from RCC and VAs from LCC, L-RCC. Last, LCC VAs could be differentiated from L-RCC VAs by using the QS duration in aVR. For patients referred for catheter ablation of LVOT VAs, this simple ECG measurement might be performed to help plan an ablation strategy and to enhance patient counseling with regard to potential outcome, procedure risk associated with mapping and ablation. 

Anatomically, the aortic root occupies a central location within the heart, and the RVOT is located anteriorly and leftward to the aortic root, with the AMC and LV summit anterosuperior to the aortic root. The AMC and the LV summit face each other at the basal portion of the LV muscle between them [5,12]. The aortic root consists of three sinuses of Valsalva. The RCC and LCC are connected with the ventricular musculature at their bases, and the NCC is located between the right and the left atria, immediately anterior to the interatrial septum [13,14]. It has been proven that ventricular myocardial extensions into the aorta beyond ventriculo-arterial junction and these myocardial extensions are the target of ablation [17]. AMC consists of the aortic annulus and the anterior side around the mitral annulus. Although this is a fibrous structure, it is known to be an arrhythmogenic site for ventricular tachyarrhythmias in both normal and diseased heart [18,19]. The LV summit constitutes the superior-most aspect of the LV ostium. It is defined as a triangular region, the apex of which is formed by the bifurcation of the left anterior descending and left circumflex arteries, with its base formed by the arc between the first septal perforator of the left anterior descending artery and the left circumflex artery [5]. The VAs originating from the LV summit can be ablated through the great cardiac vein [5,20].

The ECG characteristics of LVOT VAs are very similar and may even overlap because they all originate from adjacent cardiac structures [21]. It is important to differentiate the ECG characteristics of LVOT VAs in order to safely perform radiofrequency catheter ablation. Ouyang et al. [22] found that a great R wave duration and R/S wave amplitude ratio in lead V1 or V2 reliably predicted an aortic sinus cusp compared with right ventricular outflow tract origin. Lin et al. [23] found that the LCC VAs typically produce a precordial transition by V2, whereas RCC VAs demonstrated a precordial transition by lead V3 and precordial transition is a simple criterion for distinguishing VAs from LCC to RCC. Chen et al. [18] found that AMC VAs showed an early R/S transition pattern in the precordial leads, and VAs from anterior AMC demonstrated equal R and S amplitudes in V2, rS in V1, and R in V3, while VAs from the middle part of the AMC demonstrated a special (‘rebound’) transition pattern, with which equal R and S amplitudes occurred in V2, and high R waves in V1 and V3. However, one shortcoming of the R wave transition in the precordial leads is the QRS morphology that could alter markedly with an incorrect electrode placement [15]. Komatsu et al. [24] found that The R-wave amplitude ratio in lead III/II, Q-wave amplitude ratio in leads aVL/aVR, and ratio of R wave/S wave in lead V1 were higher in LV summit VAs than in the RVOT and the RCC VAs, the ECG characteristics of LV summit VAs were similar to those in LCC VAs, except for the presence of an initial Q wave in lead I, an initial R wave in lead V1. 

In the present study, we have demonstrated that the QS duration in aVL was significantly increased in VAs from AMC and LV summit than in VAs from aortic root, and the QS duration in aVL > 134 ms could be used to distinguish VAs from AMC, LV summit and VAs from aortic root. The R duration in II increased more in the LV summit group than in the AMC group. Anatomically, the aortic root, AMC and LV summit area lie in a continuum from the medial to the lateral, and the QRS duration of VAs from aortic root, AMC and LV summit would be increased gradually as demonstrated in our study because of synchronous rather than sequential ventricular activation [11]. For differentiating VAs from aortic root, we have found that the ratio of III/II was significantly decreased in VAs from RCC compared to VAs from L-RCC and LCC. The LCC typically sits slightly superior to the RCC. As a result, the VAs from LCC have more of an inferior axis relative to the VAs from RCC [23]. We also found that the QS duration of aVR increased in VAs from LCC than in VAs from L-RCC, and the QS duration of aVR > 130 ms can be used to distinguishing VAs from these two sites. 

To the best of our knowledge, this study is the first to investigate the surface ECG characteristics of VAs from different sites of LVOT. We found that patients with VAs from different sites of LVOT demonstrated distinct ECG characteristics, and we also presented a simple four-stepwise ECG algorithm with satisfactory sensitivity and specificity to distinguish VAs from different sites of LVOT. The utility of this ECG algorithm to predict the precise origin of LVOT VAs was validated in a prospective cohort, and the algorithm also exhibited a good predictive value in discriminating LVOT VAs. The last step of the algorithm showed relatively low sensitivity and specificity; this would be explained as the population of our study was small, LCC and L-RCC are closely related, and it is difficult for ECG to distinguish VAs from these areas. Our algorithm uses limb leads, which are less influenced by lead position. Moreover, based upon the duration and amplitude of the QRS wave, the algorithm is simple and practicable in routine clinical practice to help plan an ablation strategy and to enhance patient counseling. 

### Study Limitations

There were several limitations in the present study. First, the study population was relatively small, which might result in statistical bias, and the results of our study should be validated further in larger populations. Second, the origins of LOVT VAs were defined by the successful ablation sites, which might not be the real origin but anatomically in close proximity to it. Furthermore, the patients requiring multiple sites ablation were excluded from this study, and these may lead to subjective bias. Third, cases with structural heart disease that affect cardiac anatomy were excluded, and the stepwise ECG algorithm would not be suitable for patients with structural heart disease. Fourth, intracardiac echocardiography was not used, only coronary angiography and 3-dimensional electroanatomic mapping were used to determine the final ablation sites; sometimes it is difficult for us to distinguish LCC-RCC VAs from LCC VAs. Finally, the long-term success was not confirmed in this study, as there might be late recurrence cases in these subjects.

## 5. Conclusions

We have developed a simple four-stepwise ECG algorithm to discriminate the AVs from different sites of LVOT, and our algorithm demonstrated good predictive value in the prospective cohort. Further studies are warranted to verify our results.

## Figures and Tables

**Table 1:** Patients and clinical characteristics.

Characteristic	RCC Group (*n* = 8)	L-RCC Group (*n* = 21)	LCC Group (*n* = 24)	AMC Group (*n* = 9)	LV Summit Group (*n* = 6)
Age (year)	45 ± 19	50 ± 16	56 ± 16	66 ± 15	54 ± 20
Gender (male)	1 (13%)	7 (33%)	11 (46%)	5 (56%)	4 (67%)
BMI (kg/m^2^)	24.9 ± 4.8	26.3 ± 6.2	25.2 ± 5.3	27.0 ± 7.1	25.3 ± 4.8
Hypertension (n,%)	3 (37%)	7 (33%)	9 (37%)	4 (44%)	2 (33%)
Diabetes	1 (14%)	2 (9%)	3 (14%)	1 (11%)	0
LVd (mm)	45 ± 5	48 ± 4	50 ± 5	51 ± 3	49 ± 3
LVEF (%)	59 ± 3	65 ± 6	61 ± 5	61 ± 6	61 ± 7
History (month)	17 ± 13	21 ± 28	30 ± 31	30 ± 26	65 ± 58 *^#^
PVC burden (n/24 h)	20,653 ± 13,707	23,279 ± 12,299	22,302 ± 8208	23,751 ± 11,356	34,843 ± 13,899
VT (%)	1 (13%)	2 (9%)	2 (8%)	0	2 (33%)
Antiarrhythmics (n)	2.1 ± 1.1	1.9 ± 1.3	2.0 ± 1.2	2.2 ± 1.2	1.8 ± 1.3

Values are presented as mean ± SD or as n (%). BMI, body mass index; LVd, left ventricular diameter; LVEF, left ventricular ejection fraction; PVC, premature ventricular contraction; VT, ventricular tachycardia. * *p* < 0.05 compared with RCC group; ^#^ *p* < 0.05 compared with L-RCC group.

**Table 2:** Comparison of ECG characteristics of limb leads.

Characteristic	RCC Group (*n* = 8)	L-RCC Group (*n* = 21)	LCC Group (*n* = 24)	AMC Group (*n* = 9)	LV Summit Group (*n* = 6)
Total QRS duration (ms)	150 ± 28	142 ± 15	147 ± 13	158 ± 13 ^#^	178 ± 26 *^#&$^
R wave duration (ms)					
Lead I	100 ± 35	88 ± 33	83 ± 35	73 ± 26	103 ± 41
Lead II	132 ± 13	134 ± 16	142 ± 14	152 ± 11 *^#^	171 ± 30 *^#&$^
Lead III	131 ± 11	129 ± 19	139 ± 16	152 ± 11 *^#^	169 ± 34 *^#&^
Lead aVF	130 ± 10	133 ± 17	141 ± 16	151 ± 11 *^#^	168 ± 30 *^#&^
QS wave duration (ms)					
Lead I (S wave)	35 ± 7	59 ± 23	64 ± 27	81 ± 29	71 ± 46
Lead aVL	113 ± 19	124 ± 18	130 ± 17 *	147 ± 13 *^#&^	153 ± 24 *^#&^
Lead aVR	125 ± 14	128 ± 14	139 ± 15 *^#^	146 ± 14 *^#^	155 ± 15 *^#&^
R wave amplitude (mV)					
Lead I	0.46 ± 0.27	0.43 ± 0.30	0.32 ± 0.22	0,20 ± 0.11	0.29 ± 0.21
Lead II	1.75 ± 0.33	2.07 ± 0.81	2.11 ± 0.57	1.71 ± 0.28	1.84 ± 0.53
Lead III	1.40 ± 0.45	1.95 ± 0.86	2.18 ± 0.69 *	1.89 ± 0.39	1.93 ± 0.55
Lead aVF	1.60 ± 0.42	2.05 ± 0.89	2.14 ± 0.59 *	1.79 ± 0.34	1.86 ± 0.53
The ratio of III/II	0.78 ± 0.15	0.94 ± 0.16*	1.03 ± 0.13 *	1.10 ± 0.16 *^#^	1.06 ± 0.10 *
QS wave amplitude (mV)					
Lead I (S wave)	0.07 ± 0.03	0.22 ± 0.13	0.31 ± 0.17	0.39 ± 0.11	0.24 ± 0.16
Lead aVL	0.58 ± 0.29	1.14 ± 0.53 *	1.24 ± 0.35 *	1.12 ± 0.21 *	1.12 ± 0.30 *
Lead aVR	1.02 ± 0.18	1.09 ± 0.42	1.09 ± 0.21	0.79 ± 0.15 ^#&^	0.96 ± 0.25
The ratio of aVL/aVR	0.58 ± 0.28	1.12 ± 0.36 *	1.20 ± 0.36 *	1.45 ± 0.38 *^#^	1.18 ± 0.18 *

Values are presented as mean ± SD or as n (%). * *p* < 0.01 compared with RCC group; ^#^ *p* < 0.01 compared with L-RCC group; ^&^ *p* < 0.01 compared with LCC group; ^$^ *p* < 0.05 compared with AMC group.

**Table 3:** Comparison of ECG characteristics of precordial leads.

Characteristic	RCC Group (*n* = 8)	L-RCC Group (*n* = 21)	LCC Group (*n* = 24)	AMC Group (*n* = 9)	LV Summit Group (*n* = 6)
R wave duration (ms)					
Lead V1	46 ± 10	52 ± 24	72 ± 32 *	101 ± 52 *^#&^	134 ± 17 *^#&^
Lead V2	54 ± 14	72 ± 33	88 ± 27	113 ± 35 *^#&^	127 ± 12 *^#&^
Lead V3	92 ± 25	108 ± 24	112 ± 23 *	124 ± 31 *	132 ± 18 *
Lead V4	129 ± 15	127 ± 16	131 ± 14	137 ± 23	148 ± 22 *^#&^
Lead V5	134 ± 14	130 ± 14	135 ± 14	139 ± 22	150 ± 23
Lead V6	113 ± 41	130 ± 13	134 ± 12 *	142 ± 16 *	147 ± 28 *
S wave duration (ms)					
Lead V1	79 ± 23	69 ± 23	60 ± 23	30 ± 1 *^#&^	44 ± 7 *
Lead V2	73 ± 18	62 ± 29	56 ± 23	44 ± 13 *	43 ± 6 *
Lead V3	45 ± 15	47 ± 21	40 ± 21	37 ± 12	42 ± 15
R wave amplitude (mV)					
Lead V1	0.22 ± 0.07	0.34 ± 0.24	0.53 ± 0.42 *	0.68 ± 0.57 *^#^	0.83 ± 0.26 *^#^
Lead V2	0.45 ± 0.17	0.98 ± 0.65	1.07 ± 0.58	1.22 ± 0.76	1.47 ± 0.58
Lead V3	0.93 ± 0.32	1.57 ± 0.97	1.85 ± 0.83 *	1.77 ± 0.71 *	2.42 ± 0.94 *^#^
Lead V4	1.79 ± 0.53	2.06 ± 1.11	2.25 ± 0.68	1.84 ± 0.52	2.68 ± 0.88
Lead V5	1.92 ± 0.30	2.10 ± 0.88	2.18 ± 0.68	1.33 ± 0.43 ^#&^	2.28 ± 0.89 ^$^
Lead V6	1.75 ± 0.33	1.84 ± 0.67	1.77 ± 0.56	0.91 ± 0.34 *^#&^	1.22 ± 0.37 ^#&^
S wave amplitude (mV)					
Lead V1	1.28 ± 0.67	1.00 ± 0.58	0.61 ± 0.48 *^#^	0.16 ± 0.11 *^#^	0.37 ± 0.28 *
Lead V2	1.36 ± 0.61	1.25 ± 0.86	1.12 ± 0.66	0.48 ± 0.35 *^#&^	0.50 ± 0.16 *
Lead V3	0.55 ± 0.43	0.69 ± 0.49	0.81 ± 0.52	0.58 ± 0.51	0.45 ± 0.17

Values are presented as mean ± SD or as n (%). * *p* < 0.01 compared with RCC group; ^#^ *p* < 0.01 compared with L-RCCgroup; ^&^ *p* < 0.01 compared with LCC group; ^$^ *p* < 0.05 compared with AMC group.

**Table 4:** The accuracy of ECG algorithm for predicting the origins of VAs from LVOT in prospective cohort.

	Sensitivity, %	Specificity, %	PPV, %	NPV, %
The QS duration in aVL > 134 ms for differentiating VAs from AMC, LV summit and VAs from aortic root	100.0	86.7	76.5	100.0
The R duration in II > 155 ms for differentiating VAs from AMC and VAs from LV summit	75.0	80.0	85.7	66.7
The ratio of III/II < 0.9 for differentiating VAs from RCC and VAs from L-RCC, LCC	100.0	95.0	85.7	100.0
The QS duration in aVR > 130 ms for differentiating VAs from LCC and VAs from L-RCC	66.7	62.5	72.7	55.6

## Data Availability

The data underlying this article will be shared upon reasonable request to the corresponding author.
